# Isoform-level analyses of 6 cancers uncover extensive genetic risk mechanisms undetected at the gene-level

**DOI:** 10.1038/s41416-025-03141-y

**Published:** 2025-08-07

**Authors:** Yung-Han Chang, Sean T. Bresnahan, S. Taylor Head, Tabitha A. Harrison, Yao Yu, Chad D. Huff, Bogdan Pasaniuc, Sara Lindström, Arjun Bhattacharya

**Affiliations:** 1 Quantitative Sciences Program, University of Texas MD Anderson Cancer Center UTHealth Houston Graduate School of Biomedical Sciences, Houston, TX USA; 2 Department of Epidemiology, University of Texas MD Anderson Cancer Center, Houston, TX USA; 3 Department of Epidemiology, School of Public Health, University of Washington, Seattle, WA USA; 4 Department of Genetics, Perelman School of Medicine, University of Pennsylvania, Philadelphia, PA USA; 5 Public Health Sciences Division, Fred Hutchinson Cancer Center, Seattle, WA USA; 6 Institute for Data Science in Oncology, University of Texas MD Anderson Cancer Center, Houston, TX USA

**Keywords:** Cancer genomics, Cancer genetics, Gene regulation

## Abstract

**Background:**

Integrating genome-wide association study (GWAS) and transcriptomic datasets can identify mediators for genetic risk of cancer. Traditional methods often are insufficient as they rely on total gene expression measures and overlook alternative splicing, which generates different transcript-isoforms with potentially distinct effects.

**Methods:**

We integrate multi-tissue isoform expression data from the Genotype Tissue-Expression Project with GWAS summary statistics (all *N* > ~20,000 cases) to identify isoform- and gene-level associations with six cancers (breast, endometrial, colorectal, lung, ovarian, prostate) and six related cancer subtype classifications (*N* = 12 total).

**Results:**

Directly modeling isoforms using transcriptome-wide association studies (isoTWAS) significantly improves discovery of genetic associations compared to gene-level approaches, identifying 164% more significant associations (6163 vs. 2336) with isoTWAS-prioritized genes enriched 4-fold for evolutionarily-constrained genes. isoTWAS tags transcriptomic associations at 52% more independent GWAS loci across the six cancers. Isoform expression mediates an estimated 63% greater proportion of cancer risk SNP heritability compared to gene expression. We highlight several isoTWAS associations that demonstrate GWAS colocalization at the isoform level but not at the gene level, including *CLPTM1L* (lung cancer), *LAMC1* (colorectal), and *BABAM1* (breast).

**Conclusion:**

These results underscore the importance of modeling isoforms to maximize discovery of genetic risk mechanisms for cancers.

## Introduction

Over the past twenty years, genome-wide association studies (GWASs) have successfully linked hundreds of genetic variants associated with increased risks for common cancers, including breast, prostate, lung, and colorectal cancers [1]. For example, Zhang and Ahearn et al. identified 32 new loci associated with breast cancer risk in European ancestry individuals [2], totaling over 200 loci identified for overall and subtype-specific breast cancer risk. Similarly, Wang and Shen et al. identified 187 novel risk associations in a multi-ancestry prostate cancer GWAS [3]. Despite these findings underscoring the potential of GWASs to uncover genetic risk factors for cancer, a significant challenge remains as most genome-wide significant variants are in non-coding regions of the human genome, making it difficult to understand the biological mechanisms underlying these genetic associations.

To address this gap, transcriptome-wide association studies (TWASs) have emerged as a powerful complementary approach, providing valuable insights into the functional impact of genetic variants [4–8]. By focusing on the regulatory effects of genetic variants on gene expression, TWASs can identify potential target genes and pathways involved in disease processes. This integrated approach enhances our ability to translate genetic findings from GWASs into a deeper molecular understanding of disease etiology, which is necessary for improved diagnostics, prevention strategies, and therapeutic interventions in oncology.

Despite the promise of TWASs and related approaches, recent work suggests that gene expression may be a poor mediator of genetic risk of complex traits, potentially owing to differences in evolutionary pressures on genetic variants with large effects on complex traits and those with large effects on molecular phenotypes [9, 10]. It is possible that part of this poor mediation is due to an overreliance on total gene expression as a fundamental unit of measure of the transcriptome. Total gene expression does not account for alternative splicing that can produce multiple isoforms from the same gene, which are often under subtle genetic or environmental control. The mRNA and protein isoforms generated through alternative processing of primary RNA transcripts can vary in function and potentially affect cancer risk in different ways [11, 12]. To address this limitation, we applied isoform-level TWAS (isoTWAS), a framework that integrates genetic and isoform-level transcriptomic variation with GWAS summary statistics. This approach has previously demonstrated strong performance in increasing the discovery of brain-relevant traits, identifying transcriptomic association at far more GWAS loci compared to traditional gene-level TWAS while controlling the false discovery rate [13, 14].

In this study, we utilized isoTWAS to assess the association between isoform expression and risk of six cancer types (breast [15, 16], endometrial [17], colorectal [18], lung [19], ovarian [20], and prostate [21]), and their subtype classifications where applicable. We used publicly available European-ancestry cancer GWAS summary statistics and multi-tissue isoform- and gene-level expression data from the Genotype Tissue-Expression Project (GTEx, *N* > 100) [22]. isoTWAS reveals more associated loci than traditional TWAS, highlighting its potential to uncover novel genetic insights and enhance our understanding of cancer biology.

## Results

**Fig. 1:**
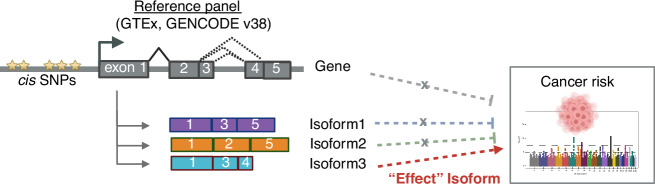
Importance of isoform expression analysis in cancer risk association. When examining only total gene expression, the gene shows no significant association with the cancer risk. However, analyzing expression at the isoform level reveals that a specific isoform of the gene are associated with the trait. This suggests that isoform-level mediation analysis can uncover regulatory mechanisms and prioritize genes that would otherwise be overlooked in total expression analyses. Created with BioRender.com.

We conducted isoform- (isoTWAS) and gene-level transcriptome-wide association studies (TWAS) for a total of 12 cancer outcomes: (1–3) breast (BRCA; overall, estrogen receptor (ER) + , ER-) [15, 16], (4) colorectal (CRC) [18], (5) lung (LUNG; overall) [19], (6) lung adenocarcinoma (LUAD) [19], (7) lung squamous cell carcinoma (LUSC) [19] (8–9) ovarian (OVCA; overall, serous) [20], (10–11) prostate (PRCA; overall, advanced) [21], and (12) endometrial (UCEC) [17]. We integrated GWAS summary statistics (*N* = 63,053–228,951) with multi-tissue expression QTL data from individuals without cancer collected post-mortem from the Genotype Tissue-Expression Project (GTEx, *N* = 108–574) (Fig. 1). We selected these 12 cancer subtypes based on their large GWAS sample sizes, particularly with sufficient case numbers, which are essential for ensuring robust and reliable downstream analyses. GWAS and GTEx sample sizes and assignments of relevant tissues to cancer outcomes are provided in Supplemental Tables 1, 2. See Methods, Supplemental Methods, Supplemental Fig. 1 for further details.

### Isoform-level analysis reveals cancer risk associations not detected at the gene-level

**Fig. 2:**
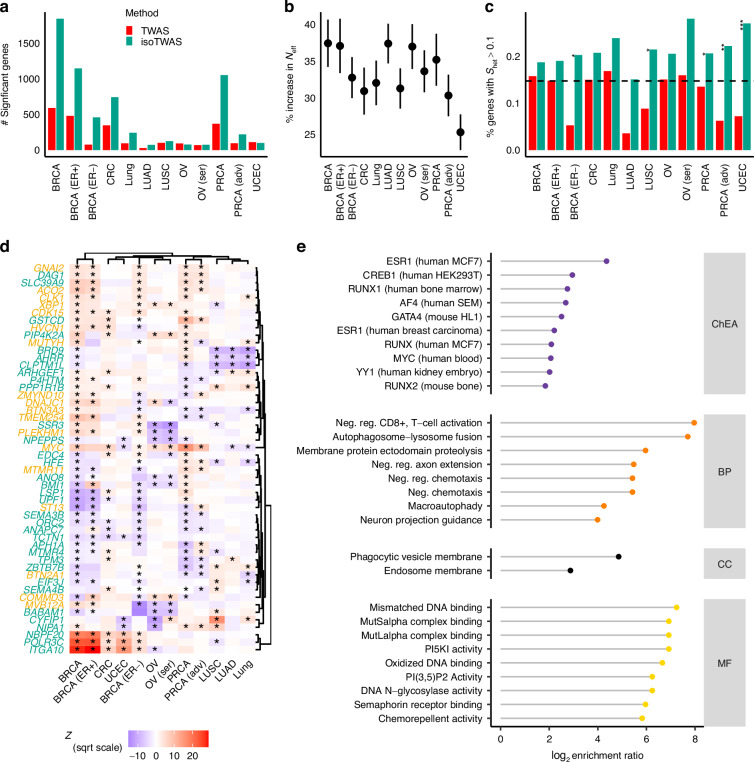
Isoform-level analysis identifies substantially more gene associations with cancer risk across 12 outcomes. **a** The number of unique transcriptome-wide significant genes identified with TWAS (red) and isoTWAS (green). **b** Percent increase in effective sample size with Wald-type 95% confidence interval using jackknife standard errors. **c** Proportion of transcriptome-wide significant genes identified with s_het_ > 0.1. Asterisks indicate FDR-adjusted *P*-value of χ2 test for enrichment ratio of high pLI genes among all transcriptome-wide significant genes across method. (*) indicates FDR-adjusted *P* < 0.05, (**) *P* < 0.01, (***) *P* < 0.005. Black line shows the genome-wide proportion of genes with s_het_ > 0.1. **d** Scaled gene-level isoTWAS Z-score for 52 genes with isoform-level risk associations with at least 5 cancer outcomes across 12 outcomes. Genes are marked in green if no gene-level risk associations with any cancer outcome, and asterisk is shown if the gene association is significant for the given cancer outcome. **e** Log-enrichment ratio (X-axis) of over-represented gene ontologies (Y-axis), across ChIP- seq identified transcription factor targets (ChEA), biological process (BP), cellular component (CC), or molecular function (MF) pathways for 34 genes with isoform-level risk associations with at least 5 cancer outcomes and no TWAS associations.

In total, isoTWAS identifies 11,078 significant isoform associations across 6163 unique genes, representing a ~164% increase in gene identification as compared to traditional TWAS which identified 2336 significant gene associations (Fig. 2a**;** Supplemental Fig. 2**;** Supplemental Tables S3–5, Supplemental Data S1, 2). Of the genes identified, 10% from isoTWAS and 7% from traditional TWAS are included in the OncoKB Cancer Gene List, representing a moderate enrichment of hallmark cancer genes among isoTWAS gene (*P* = 0.03) [23, 24]. For both TWAS and isoTWAS, most identified gene associations are observed for BRCA, ER + BRCA, and PRCA, which also are the three largest GWAS. Specifically, isoTWAS identifies 3.12 times as many associations for BRCA (3889 isoforms), 2.85 times more for PRCA (1849 isoforms), and 2.39 times as many for ER + BRCA (2,304 isoforms) as compared to TWAS, while showing relatively similar performance for LUNG; Manhattan plots of TWAS and isoTWAS results are shown in Supplemental Figs. S3–S14. Given that the mean of the χ2 distribution is linearly related to power and sample size, the percent increase in the test statistic serves as a measure of power or effective sample size. For χ2 > 1, we calculated the percent increase for isoTWAS-based associations compared to TWAS-based associations (Fig. 2b). Across 12 cancer outcomes, isoTWAS shows an average 25.3–37.4% increase in effective sample size compared to TWAS, suggesting that isoTWAS achieves comparable or greater power than TWAS, while requiring approximately 25–37% fewer samples (See Methods and Supplemental Methods for further details). Several key biological pathways are enriched for cancer-specific sets of isoTWAS-prioritized genes, including cell cycle and mitosis regulation, DNA and RNA binding, immune pathways, as well as downstream targets of cancer-relevant transcription factors like *ESR1* [25], *RUNX2* [26], and *YY1* [27] (Supplemental Table S6, Supplemental Figs. S15–S20).

We further explored if cancer risk genes identified by TWAS and isoTWAS capture true disease signals by identifying genes under selective constraint. If a gene is constrained, selection will act to remove variants that diminish gene function from the population, such as loss-of-function (LOF) variants. Here, we used Bayesian estimates of the s_het_ measure of constraint [28], which, unlike traditional measures of constraint, is not biased towards longer genes. In total, 19.9% of isoTWAS gene associations (1226 of 6163) show s_het_ > 0.1, compared to 12.5% of TWAS gene association (293 of 2336) with s_het_ > 0.1. Not only does this represent a significant enrichment of high s_het_ among isoTWAS-prioritized gene associations compared to TWAS-prioritized gene associations (χ2 test *P* = 3.7 × 10^−^^15^), isoTWAS-prioritized genes are significantly enriched compared to the genome-wide proportion of high s_het_ genes (14.7%, χ2 *P* < 2.2 × 10^−^^16^). In addition, we find significant enrichments of high s_het_ genes among transcriptome-wide significant genes for ER- BRCA, LUNG, LUSC, PRCA, Adv PRCA, and UCEC (χ2 FDR-adjusted *P*-value < 0.05; Fig. 2c, Supplemental Table S4).

Lastly, isoTWAS identifies 52 genes (34 undetected by TWAS) that are associated with five or more cancer outcomes (Fig. 2d), including multiple known oncogenes or tumor suppressor genes, such as *MYC* [29], *MUTYH* [30], *GNAI2* [31, 32], *ACO2* [33], and *BMI1* [34]. In comparison, TWAS identified 15 such genes, with four genes shared between the two methods. The 34 genes undetected by TWAS are enriched for multiple salient pathways: regulation of CD8-positive and T cells, regulation of membrane protein complexes, and phagocytic and autolysosome function. Additionally, these 34 genes are highly enriched for downstream targets of crucial oncogenic transcription factors, like *ESR1*, *GATA4*, *YY1*, and *MYC*, all of which were determined using ChIP-Seq experiments in human or mouse cancer cells or tumors (Fig. 2e) [35]. We also observed that for many genes, the directions of isoform-level effects vary across traits (Supplemental Figure 21). Genes associated with a larger number of cancers tend to show greater expression variability across tissues. For many of these genes, such as *MYC*, distinct isoforms can show opposing effects (Supplemental Figure 22). Altogether, not only can isoTWAS identify constrained susceptibility genes for multiple cancers, but it can also reveal pan-cancer risk signals that TWAS is unable to detect, possibly due to opposing directions of effects of two or more isoforms of the same gene.

### Isoform expression explains more GWAS loci and overall SNP heritability of cancer risk

**Fig. 3:**
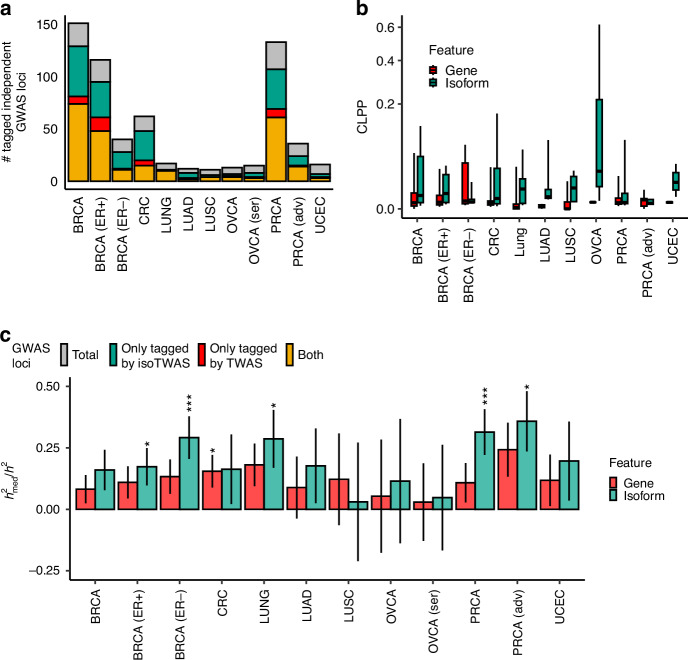
Isoform expression potentially mediates far more GWAS signal than gene expression. **a** The number of independent GWAS loci tagged by TWAS (red) or isoTWAS (green) or both in common (gold). **b** Boxplots of CoLocalization Posterior Probability (CLPP, Y-axis, square-root axis) of GWAS and gene expression QTL (red) and isoform expression QTL (green) for genomic loci with a GWAS *P* < 5 × 10^−^^8^ and QTL *P* < 10^−^^6^. **c** Ratio of gene- (red) and isoform-level (green) expression mediated heritability (h^2^_med_) and total SNP heritability (h^2^) with standard errors. Asterisks indicate FDR-adjusted *P*-value of Wald-type Z tests of h^2^_med_/h^2^ = 0. (*) indicates FDR-adjusted *P* < 0.05, (**) *P* < 0.01, (***) *P* < 0.005.

In addition to increasing the discovery of susceptibility genes across the genome, isoTWAS can identify far more transcriptomic mechanisms within independent, high-confidence genome-wide significant loci. Across all cancer outcomes, we identify 622 risk-associated GWAS SNPs (*P* < 5 × 10^−^^8^) in independent linkage disequilibrium (LD) blocks (see Methods). Among these 622 loci, 288 (46.3%) are tagged by TWAS, and 439 (70.6%) are tagged by isoTWAS, with 249 (40.0%) loci identified by both TWAS and isoTWAS (Fig. 3a, Supplemental Table S7). In total, this represents an increase in significant associations in GWAS loci of 52.4% when using isoTWAS, rather than TWAS. Additionally, isoTWAS identified 2911 unique significant genes outside of known GWAS loci.

We also considered an orthogonal analysis to compare GWAS follow-up using both gene and isoform expression. We mapped *cis*-expression quantitative trait loci (eQTLs) for gene and isoform expression and conducted Bayesian colocalization analysis with GWAS signals using eCAVIAR [36] to estimate the CoLocalization Posterior Probability (CLPP; see Methods; Supplemental Table S8, Supplemental Data S3). Generally, isoTWAS exhibits higher CLPPs compared to TWAS, indicating a stronger likelihood of colocalization for isoform-eQTLs with GWAS loci (Fig. 3b), with the proportion of loci having isoform expression Quantitative Trait Loci (isoQTL) colocalizations with CLPP > 0.01 consistently higher across the 12 cancer outcomes (Supplemental Figure S23). Notably, in OVCA and UCEC, isoTWAS shows significantly higher CLPPs (median = 0.026, *P* = 0.013) than TWAS (median = 0.001, *P* = 0.001), suggesting that isoTWAS more effectively captures colocalized signals in for these cancer outcomes. Additionally, we estimated the proportion of total SNP heritability (h^2^) mediated by gene- and isoform-level expression (h^2^_med_), (Fig. 3c and Supplemental Table S9). Overall, isoform-level expression explains 62.7% more of cancer risk SNP heritability (19.2 ± 10.3%) compared to gene-level expression (11.8 ± 5.7%). Wald-type tests reveal that isoform expression mediates a significant proportion of cancer h^2^, with gene expression only explaining a significant portion for ER- BRCA (isoTWAS: 0.291, FDR-adjusted *P* = 0.005; TWAS: 0.133, *P* = 0.068), ER + BRCA (isoTWAS: 0.173, *P* = 0.045; TWAS: 0.110, *P* = 0.102), LUNG (isoTWAS: 0.287, *P* = 0.044; TWAS: 0.181, *P* = 0.056), PRCA (isoTWAS: 0.314, *P* = 0.005; TWAS: 0.108, *P* = 0.164), and Adv PRCA (isoTWAS: 0.358, *P* = 0.014; TWAS: 0.243, *P* = 0.046). These results indicate that not only does isoTWAS recapitulate an overwhelming majority of TWAS signals at GWAS loci, isoTWAS substantially increases discovery of candidate GWAS mechanisms and transcriptomic features that potentially mediate genetic effects on cancer risk.

### isoTWAS and isoform-eQTL colocalization prioritize undetected mechanisms at GWAS loci

**Fig. 4:**
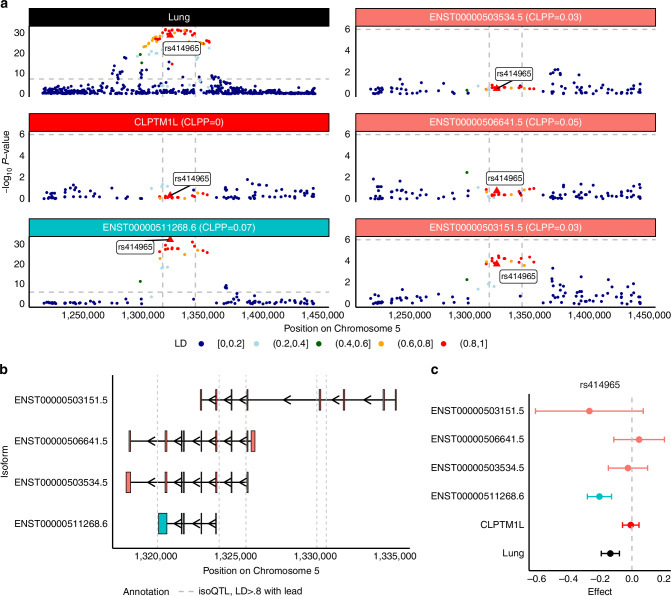
CLPTM1L isoforms may mediate lung cancer risk GWAS locus at Chromosome 5p15.33. **a** Manhattan plot of GWAS effects, *CLPTM1L* gene-eQTLs, and isoform-eQTLs for all significantly associated isoforms of *CLPTM1L*, colored by LD to rs414965, the lead isoQTL for ENST00000511268.6. **b** Transcript structure of significantly associated isoforms of *CLPTM1L*. Vertical lines indicated significant isoQTLs of LD > 0.8 to rs414965, strongest isoQTL of *CLPTM1L* isoforms. **c** SNP effect sizes on lung cancer risk (black), *CLPTM1L* gene expression (red), ENST00000511268.6 isoform expression (blue), and other expression of other isoforms (peach) for rs414965.

**Fig. 5:**
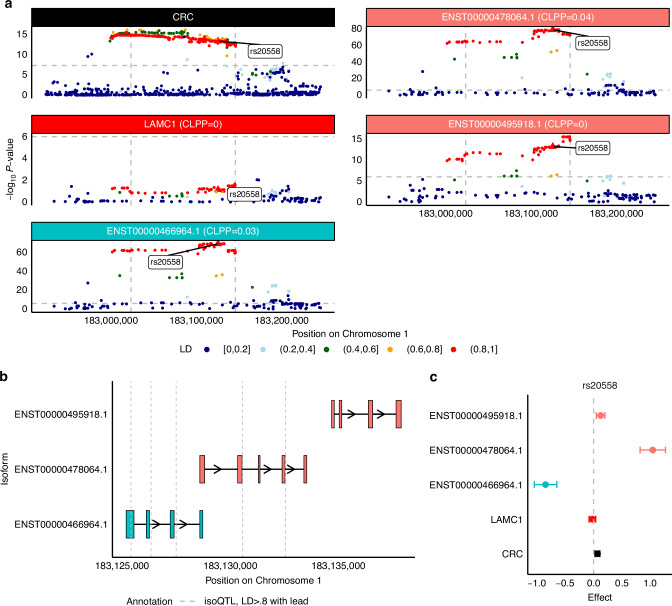
LAMC1 isoforms may mediate colorectal cancer GWAS locus at Chromosome 1q25.3. **a** Manhattan plot of GWAS effects, *LAMC1* gene-eQTLs, and isoform-eQTLs for all significantly associated isoforms of *LAMC1*, colored by LD to rs20558, the lead isoQTL for ENST00000466964.1. **b** Transcript structure of significantly associated isoforms of *BABAM1*. Vertical lines indicated significant isoQTLs of LD > 0.8 to rs20558, strongest isoQTL of *LAMC1* isoforms. **c** SNP effect sizes on colorectal cancer risk (black), *LAMC1* gene expression (red), ENST00000466964.1 isoform expression (blue), and other expression of other isoforms (peach) for rs20558.

**Fig. 6:**
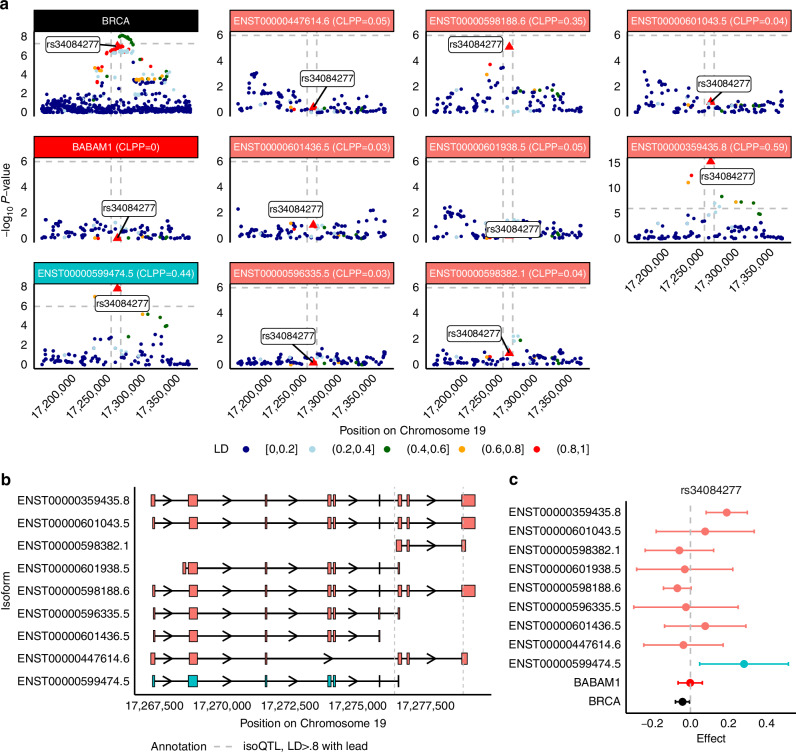
BABAM1 isoforms may mediate breast cancer risk GWAS locus at Chromosome 19p13.11. **a** Manhattan plot of GWAS effects, *BABAM1* gene-eQTLs, and isoform-eQTLs for isoforms of *BABAM1*, either prioritized through isoTWAS or with an isoQTL with *P* < 1e-6. **b** Transcript structure of *BABAM1*. Vertical lines indicated significant isoQTLs of LD > 0.8 to rs34084277, strongest isoQTL of *BABAM1* isoforms. **c** SNP effect sizes on breast cancer risk (black), *BABAM1* gene expression (red), expression of isoTWAS-prioritized isoforms (blue), and expression of other isoforms (peach) for rs34084277 (lead isoQTL).

A main goal of isoform-specific analyses is to nominate a more granular hypothesis of transcriptomic regulation. Post-hoc transcript-level fine-mapping reveals nine loci where isoTWAS prioritization coincides with isoform-eQTL colocalization (CLPP > 0.01) but no gene-eQTL colocalization (Methods; Supplemental Data S3), across BRCA (Fig. 4, Supplemental Fig. S24), ER- BRCA (Supplemental Figs. S25–S26), CRC (Fig. 5, Supplemental Fig. S27), LUNG (Fig. 6), PRCA (Supplemental Fig. S28), and UCEC (Supplemental Fig. S29). We highlight *CLPTM1L*, *LAMC1*, and *BABAM1* here due to previously-reported pleiotropic associations across multiple cancers. *CLPTM1L* is located near the *TERT* locus, known for its pleiotropic links to various cancers, including ER- BRCA, CRC, glioma, LUNG, melanoma, OVCR, pancreatic, and PRCA cancer [37], the *LAMC1* locus has shown associations with UCEC, glioma, and PRCA [38–40], and *BABAM1* is a recognized GWAS hit with broad implications for cancer risk, including its interaction with the well-known breast cancer-related gene *BRCA1* [41–43]. Additional examples which follow a similar pattern are presented in the Supplemental Results. We leveraged (1) functional annotations from ENCODE and ROADMAP Epigenomic Project (Supplemental Table 10), (2) splice-site QTL (sQTL) and 3’UTR alternative polyadenylation QTL (apaQTL) summary statistics from GTEx, and (3) predicted splicing potential of variants using SpliceAI [44] to investigate chromatin state and regulatory activity or more detailed splicing mechanisms near the implicated loci [22, 45–48]. For these examples, we refer to the isoform of a gene that passes all isoTWAS post-hoc analyses as the “prioritized isoform” and the top identified isoQTL by *P*-value as the isoform’s “lead isoQTL”.

First, four isoforms of *CLPTM1L* (Chromosome 5p15.33, s_het_ = 0.02, 16 total isoforms in GENCODE) are significantly associated with LUNG. We also find that isoforms of *CLPTM1L* are associated with BRCA, PRCA, LUAD, and LUSC. Fine-mapping prioritizes ENST00000511268.6 in the 90% credible set with Posterior Inclusion Probability (PIP) = 0.90. Although there are many genome-wide significant SNPs within the gene body of *CLPTM1L*, no gene-eQTL signal is observed (*P* < 10^-6^), whereas a strong ENST00000511268.6 isoform-eQTL signal colocalizes with the GWAS signal (CLPP = 0.07). Additionally, there is no strong isoform-eQTL signal for the other three isoforms identified via isoTWAS in this region. The lead isoQTL rs414965 is in high LD with multiple genome-wide significant SNPs (Fig. 4a). The exons comprising ENST00000511268.6 overlap with those of ENST00000503534.5 and ENST00000506641.5 with significant isoform-eQTLs flanking ENST00000511268.6 (Fig. 4b). However, rs414965 does not have significant effects on ENST00000503534.5, ENST00000506641.5, or ENST00000503151.5 expression (Fig. 4c), despite its strong negative associations with LUNG and ENST00000511268.6 expression. We observed a CCCTC-binding factor (CTCF) peak at the terminal exon of the prioritized isoform ENST00000511268.6 and POLR2AphosphS5 in the last exon. Additionally, we find an alternative enhancer at the 5’ end of the prioritized isoform. These findings provide functional annotation of the prioritized isoform, highlighting coordinated regulatory mechanisms that may contribute to its expression and RNA processing (Supplemental Fig. 30). ENST00000511268.6 showed strong expression in lung, spleen, and small intestine, with limited differential isoform usage across tissues, and a strong negative correlation with ENST00000503534.5 (Supplemental Fig. 31).

Next, three isoforms of *LAMC1* (Chromosome 1q25.3, s_het_ = 0.10, six total isoforms in GENCODE) are associated with CRC. Fine-mapping prioritizes only ENST00000466964.1 in the 90% credible set with PIP = 1. Again, genome-wide significant CRC-associated SNPs within the gene body show no gene-eQTL signal (*P* < 10^-6^) but a strong ENST00000466964.1 isoform-eQTL signal that colocalizes with the GWAS signal (CLPP = 0.03) was observed. For the other two isoforms, ENST00000478064.1 and ENST00000495918.1, the lead isoQTL rs20558 is in high LD with multiple genome-wide significant SNPs (Fig. 5a). The exons of these three isoforms are generally distinct sets with significant isoform-eQTLs generally falling on the 3’ end of multiple exons (Fig. 5b). rs20558 shows a significant association with increased CRC risk, no effect on *LAMC1* gene expression, and a significant decreasing effect on ENST00000466964.1 expression (Fig. 5c). Interestingly, this same SNP has a significant increasing effect on the expression of ENST00000478064.1 and ENST00000495918.1, though only ENST00000478064.1 colocalizes with CRC risk at CLPP > 0.01. One of the SNPs in perfect LD (LD = 1) with our lead isoQTL is not present in the GTEx panel we used but shows potential splicing effects based on SpliceAI predictions [44, 49]. rs34133998 may disrupt an existing donor site with stronger probability (Donor Loss = 0.77). The result supports a possible regulatory mechanism involving splicing (Supplemental Table 11). We observed splicing events that are associated with our perfect LD SNPs at the start site of the prioritized isoform ENST00000466964.1 in both subcutaneous adipose and colon tissues. We also observed an enhancer overlapping the first three exons of the prioritized isoform. The presence of POLR2A and POLR2AphosphoS5 peaks at the terminal exon provides supporting evidence for transcriptional pausing and termination at that site (Supplemental Fig. 32). We identified a relatively higher expression in two tissues—subcutaneous adipose and cultured fibroblasts—at both the gene and transcript levels, while ENST00000495918.1 exhibited high isoform usage in whole blood tissue. (Supplemental Figure 33).

Lastly, nine isoforms of *BABAM1* (Chromosome 19p13.11, s_het_ = 0.05, 18 total isoforms in GENCODE) are associated with BRCA. In our study, isoforms of *BABAM1* are also associated with OVCA, OVCA ser, LUSC, and ER- BRCA in isoTWAS. Fine-mapping prioritizes ENST00000599474.5 in the 90% credible set and a PIP of 1. There are multiple genome-wide significant BRCA-associated SNPs (*P* < 5 × 10^−8^) within the *BABAM1* gene body showing no gene-eQTL signal (*P* < 10^−^^6^). Only ENST00000599474.5 (CLPP = 0.44) and ENST00000359435.8 (CLPP = 0.59) show strong isoQTL effects that colocalize with the GWAS signal. In addition, the lead isoQTL rs34084277 for ENST00000599474.5 is in high LD (LD > 0.8) with multiple genome-wide significant SNPs (Fig. 6a). We observed two splicing events associated with the SNPs in perfect LD with the lead isoQTL in both subcutaneous adipose and breast tissue. We also observed CTCF at the terminal exon (Supplemental Figure 34). We did not observe large variance in the expression of the prioritized isoform ENST00000599474.5 across tissues. ENST00000598188.5 exhibited high isoform usage; however, none of the transcripts demonstrated clear tissue-specific differences in isoform usage. Additionally, we found a high positive correlation among six transcripts, including the prioritized isoform (Supplemental Figure 35). The exon structure of these nine isoforms reveals a group of exons at the 3’ end of the gene body, which is flanked by the lead isoQTLs of ENST00000599474.5 (Fig. 6b). Specifically, due to this unique exon structure and previous evidence of *BABAM1* harboring rare variants associated with BRCA risk [41–43], we followed up on this locus using a rare-variant analysis in UK Biobank whole exome sequencing data [50] using the Variant Annotation, Analysis, and Search Tool (VAAST2) [51, 52]. We find three BRCA-associated isoforms in *BABAM1* enriched for risk-associated rare variants (MAF < 0.5%), with associations mainly concentrated in exons at the 5’ end and first exon at the 3’ end of the transcript (Supplemental Methods; Supplemental Fig. 36; Supplemental Tables S12–S13). See Supplemental Results for further details. Lastly, rs34084277 has a strong protective effect on *BRCA* risk, no effect on *BABAM1* gene expression, and a significantly increasing effect on expression of ENST00000599474.5 and ENST00000359435.8 (Fig. 6c).

Of note, isoTWAS associations for *CLPTM1L*, *LAMC1*, and *BABAM1* were all prioritized using models trained in subcutaneous adipose tissue. To investigate why these genes are identified specifically in subcutaneous adipose, we conducted a GTEx QTL analysis for the prioritized transcripts to determine whether this is due to tissue-specific effects or simply a matter of statistical power. Supplemental Fig. 37 shows that, for the prioritized isoforms, the lead isoQTL and those SNPS in high LD also have large QTL effects in the corresponding tissue of origin, suggesting that these isoform effects may be present in other tissues but suffer from low statistical power in these tissues.

## Discussion

We show that integrating cancer risk GWAS summary statistics with isoform-level transcriptomic variation can greatly increase discovery of susceptibility genes for six cancers and their subtype classifications. By using isoTWAS rather than traditional gene-level TWAS, we identify nearly 2.5-fold more associations. More saliently, isoTWAS-identified genes are significantly enriched for evolutionarily constrained genes (s_het_ > 0.1), which previously studies have shown to be likely to contain clinically-relevant de novo or rare variants and hypothesize to predict drug toxicity and characterize transcriptional regulation [10, 53]. Isoform expression, rather than total gene expression, captures more risk-associated genetic variation and exhibits stronger colocalization with GWAS signals. Additionally, though isoform expression alone does not reconcile most of the SNP heritability of cancer risk, isoform expression is estimated to mediate nearly twice as much heritability as gene expression, and nearly three times of the SNP heritability signal in the case of PRCA. Most importantly, isoTWAS can find isoform associations at 249 of 288 GWAS loci tagged by TWAS-identified genes and uniquely tag 190 additional GWAS loci that cannot be contextualized via TWAS. In aggregate, these findings underscore the utility of considering the transcriptome on the transcript-isoform, rather than gene-level. By modeling a different quantification of the same steady-state RNA-seq datasets with sample sizes of only up to ~800, isoTWAS increases discovery specifically at GWAS loci by ~52% without additional sequencing costs.

Our isoform-level fine-mapping coupled with eQTL colocalization identifies nine gene candidates with GWAS risk SNPs within the gene body that colocalize with isoQTLs despite exhibiting no gene-eQTL signal at *P* < 10^−^^6^. We discuss three of these gene candidates. First, isoTWAS identifies an association between ENST00000511268.6, an isoform of *CLPTM1L* (16 total isoforms), and LUNG. Studies spanning back 15 years have identified multiple polymorphisms within the *CLPTM1L* gene body associated with LUNG, as well as pleiotropic associations with other malignancies [54–58]. In particular, an in vitro study provided evidence that *CLPTM1L* is oncogenic, specifically for Ras-driven lung cancers, in line with its effect on protecting tumor cells from genotoxic apoptosis [59], and is a promising therapeutic target for therapy-resistant tumors [60]. The regulatory annotation region reveals a CTCF peak at the terminal exon and POLR2AphosphoS5 signal at the last exon of the prioritized isoform ENST00000511268.6, suggesting transcription termination through polymerase stalling and recruitment of the splicing machinery, respectively. An enhancer at the 5’ end of the lead transcript, suggesting regulatory activity in this region that may influence isoform expression. This association remains inferential in the absence of direct molecular validation linking the enhancer to the isoform. In non-cancerous lung tissue, strong transcriptional activity overlaps with three transcripts of *CLPTM1L*, whereas in lung tumor, it is largely confined to the prioritized isoform, suggesting that genetic regulation of the isoTWAS-prioritized isoform is potentially cancer-specific. *CLPTM1L* (Chromosome 5p15.33) is also local to *TERT*, which harbors pleiotropic associations with multiple cancers [37]. Our study identifies associations between *CLPTM1L* and PRCA, consistent with previous findings of SNPs in the 5p15.33 region associated with PRCA [61, 62]. However, while *TERT* is known for its pleiotropic effects, we observed only gene-level associations for *TERT* in our analyses, with no isoTWAS associations detected for its specific isoforms. Further work is needed to fully interrogate this pleiotropy and assess tissue-specific isoform expression for both *TERT* and *CLPTM1L*, especially since *TERT* shows low expression across multiple tissues in GTEx [22].

We find a similar pattern for colorectal cancer and ENST00000466964.1, an isoform of *LAMC1* (six total isoforms), a gene that regulates cell adhesion, differentiation, migration, and signaling, and lies at a pleiotropic locus for CRC, PRCA [38], and obesity risk [63]. A previous study incorporating splicing measures has prioritized *LAMC1* as a novel transcriptome-mediated CRC locus [64]. Additionally, rs34295433 in *LAMC1* was identified as a susceptibility SNP for PRCA in a Taiwanese population [38]. However, in our study, isoTWAS did not detect any associations between *LAMC1* and PRCA. *LAMC1* belongs to the laminin family of extracellular matrix proteins that are significantly involved in survival and proliferation of cancer cells, angiogenesis, migration and basement membrane breach by cancer cells, and metastatic events [65]. Computational analyses of laminin proteins have shown their prognostic ability in colorectal cancer progression, placing higher weights for *LAMC1* compared to other constituents of the family [66]. One SNP in perfect LD with the lead isoQTL exhibits potential splicing disruption according to SpliceAI predictions, supporting a regulatory mechanism involving isoform-specific splicing. We observed splicing events associated with these SNPs at the start site of the prioritized isoform ENST00000466964.1 in both subcutaneous adipose and colon tissues, suggesting their potential influence on splicing regulation. Enhancer activity spanning the 5’ end of the transcript further supports a regulatory role in this region. Additionally, the presence of POLR2A and POLR2AphosphoS5 peaks at the terminal exon indicates potential transcriptional pausing and isoform-level regulatory control.

Lastly, isoTWAS detects an association between BRCA and ENST00000599474.5, an isoform of *BABAM1* (18 total isoforms), a gene involved in checkpoint signaling, regulation of DNA repair, and mitosis. *BABAM1* has previously been implicated as a low-penetrance risk locus that interacts with *BRCA1* in both triple-negative breast cancer and ovarian cancer risk [41–43]. Consistent with previous studies, isoTWAS also identifies associations within this region for OVCA, OVCA ser, and ER- BRCA. Observed splicing events associated with high-LD SNPs in both subcutaneous adipose and breast tissues further suggest a potential regulatory role in splicing. Additionally, the presence of a CTCF binding peak at the terminal exon supports the hypothesis of transcriptional termination at this region, potentially contributing to isoform-specific expression regulation. Additionally, our VAAST analysis for *BABAM1* in UKBB indicates that a group of exons at the 3’ end of the gene harbor potentially disease-causing rare variants (MAF < 0.01, only in coding regions). The lead isoQTL for our isoTWAS-prioritized isoform of *BABAM1* (MAF > 0.01, both coding and non-coding region) is upstream of these exons at the 3’ end, potentially influencing the splicing patterns specifically at the 3’ end of the gene. Further research is required to investigate how these isoQTLs influence splicing regulation and their specific role in tumorigenesis and cancer development, but a methodological opportunity may lie in integrating splicing- and isoQTLs with rare variants to identify transcriptomic mechanisms for cancer risk. These results underscore that the added resolution provided with isoform expression can lead to more specific mechanistic hypotheses for cancer risk and inform follow-up with functional studies, both in silico and experimental.

We conclude with three limitations of our work. First, the complexity of the *BABAM1* transcript structure and the sheer number of *BABAM1* isoforms with strong isoQTL signals underscores a methodological opportunity. We applied the FOCUS framework which leverages a non-informative prior that may be insufficient [67]. Fine-mapping of isoforms is challenging due to horizontal pleiotropy of SNP-isoform effects shared across exons and strong LD patterns. This horizontal pleiotropy can reduce power and increase false-positive rates. Incorporating classes of isoforms with shared exon sets may lead to improved fine-mapping, thereby increasing coverage and resolution of credible sets of isoforms within a GWAS locus.

Second, we derived isoform-level quantifications using Salmon, a method constrained by the limitations of short-read RNA-seq where maximum-likelihood estimates are based on transcriptomic annotations, introducing some uncertainty. The limited diversity of tissue-specific annotated isoforms may affect the accuracy of these estimates. In contrast, long-read RNA-seq can capture splicing and structural variation missed by short-read RNA-seq, revealing more complex and rarer transcript-isoforms. As long-read RNA-seq becomes increasingly scalable, its comprehensive view of transcriptomes will allow for more precise quantification of isoforms potentially improving isoTWAS performance. Additionally, the prioritization of these three genes in subcutaneous adipose tissue may reflect tissue-specific regulatory mechanisms, but it could also result from the statistical power across tissues, driven by sample size or the robustness of isoform quantification. Our QTL analysis indicates that the isoQTL effects observed in adipose tissue are also detectable in the tissues of origin, and the regulatory architecture appears similar between adipose and the original tissue. This may suggest that the observed isoTWAS associations in subcutaneous adipose and not the tissue of origin are likely due to power limitations rather than true tissue specificity. Long-read sequencing technologies, which offer improved isoform-level resolution, may help clarify the robustly expressed isoforms in each tissue and reveal genuine tissue-specific genetic regulation at risk loci. Third, as current eQTL datasets are mostly generated in populations of European ancestry, we focused the current analyses on cancer GWAS summary statistics based on individuals of European ancestry, as expression models trained in predominantly European-ancestry cohorts fail to predict expression accurately in individuals of different ancestries [68–71]. Future data generation should increase diversity in molecular datasets accompanied by methodological research to model multi-ancestry samples across eQTL and GWAS datasets.

## Material & Methods

### Data collection

#### GTEx genotype and transcriptomic data

For 48 tissues from GTEx [22], we quantified RNA-seq data (all *N* > 100) using Salmon v1.5.2 in mapping-based mode [72]. We built a decoy-aware transcriptomic index in Salmon with GENCODE v38 transcript sequences and the full GRCh38 reference genome as decoy sequences [45]. Salmon was then run on FASTQ files with mapping validation and corrections for sequencing and GC bias. We then imported Salmon isoform-level quantifications and aggregated to the gene-level using tximeta v1.16.1 [73]. Using edgeR, gene and isoform-level quantifications underwent TMM-normalization, followed by transformation into a log-space using the variance-stabilizing transformation using DESeq2 v1.38.3 [74, 75]. We then residualized isoform-level and gene-level expression (as log-transformed CPM) by all tissue-specific covariates (clinical, demographic, genotype principal components (PCs), and expression PEER factors) used in the original QTL analyses in GTEx.

SNP genotype calls were derived from Whole Genome Sequencing data from individuals of European ancestry, filtering out SNPs with minor allele frequency (MAF) less than 5% or that deviated from HWE at *P*  <  10^−5^. We further filtered out SNPs with MAF less than 1% frequency among the European ancestry samples in 1000 Genomes Project [76]. Due to limited sample sizes in other ancestry groups in GTEx, we focused solely on European ancestry for this study.

### GWAS summary statistics

We obtained GWAS summary statistics for risk of 12 cancer outcomes, all from samples of European-ancestry individuals [77]: overall breast cancer (BRCA; 122,977 cases/105,974 controls) [15], estrogen-receptor positive breast cancer (ER + BRCA; 69,501 cases/95,042 controls) [15], estrogen-receptor negative breast cancer (ER- BRCA; 30,882 cases/110,058 controls) [16], colorectal cancer (CRC; 55,168 cases/65,160 controls) [18], overall lung cancer (LUNG; 29,266 cases/56,450 controls) [19], lung adenocarcinoma (LUAD; 11,273 cases/55,483 controls) [19], lung squamous cell Carcinoma (LUSC; 7426 cases/55,627 controls) [19], overall ovarian cancer (OVCA; 22,406 cases/40,951 controls) [20], serous ovarian cancer (OVCA ser; 19,890 cases/68,502 controls) [20], overall prostate cancer (PRCA; 79,166 cases/61,106 controls) [21], advanced prostate cancer (Adv PRCA; 15,167 cases/58,308 controls) [21], endometrial cancer (UCEC; 12,906 cases/108,979 controls) [17]. We selected these 12 cancer subtypes based on their large GWAS sample sizes.

### Statistical analysis

#### Code and data availability

Sample scripts for analyses are available from github.com/bhattacharya-a-bt/MultiCancerIsoTWAS. Links to GWAS summary statistics are provided in Supplemental Table S1. isoTWAS and TWAS models are available from https://zenodo.org/records/11048201 [78]. GTEx genotyping and expression data were accessible from dbGaP accession number phs000424.v9. Supplemental Data Tables S1–S3 can be downloaded from https://zenodo.org/records/14010391.

### Isoform- and gene-level transcriptome-wide association studies

We trained predictive models of gene and isoform expression using all *cis*-SNPs within 1 Mb of the gene body for the 12 cancer outcomes (Supplemental Fig. 1A), using predictive models described previously^13^. We selected the best gene or isoform model through a 10-fold cross-validation and retained genes and isoforms that could be predicted at cross-validation R^2^ > 0.01. Using these predictive models, we employed a weighted burden test to estimate the association between the genetically-predicted component of a gene or isoform with cancer risk (Supplemental Fig. 1B). To account for multiple testing burden for isoform associations, we used a two-stage testing framework, as described previously^13^ (Supplemental Fig. 1C). At the end of these two steps, isoTWAS identified a set of genes and their isoforms that were associated with the trait.

For significant genes (from TWAS) and isoforms (from isoTWAS), we tested whether the SNP-gene/isoform effects from the predictive models add additional information beyond the SNP-risk effects from the GWAS through a conservative permutation test. We permuted the SNP-gene/isoform effects in the predictive models 10,000 times and generated a null distribution for the gene/isoform test statistic, which was used to calculate a *P*-value. We only conducted this permutation test for genes from TWAS with FDR-adjusted *P* < 0.05 and isoforms from isoTWAS with FDR-adjusted *P* < 0.05 and FWER-adjusted *P* < 0.05. After permutation testing, we conducted isoform- and gene-level fine-mapping for isoTWAS and TWAS associations that overlap in a 1 Mb window using methods from the FOCUS framework [67]. We accounted for the correlation between genetically-predicted isoform or gene expression induced by LD and shared prediction weights and control for certain pleiotropic effects. Through Bayesian methods, we estimated the Posterior Inclusion Probability (PIP) for each isoform or gene and defined a credible set of isoforms or genes to explain the signal with 90% confidence.

### Effective sample size

We used the mean ${\chi }^{2}$ statistic from TWAS test results to assess effective sample size. Under the alternative hypothesis, the ${\chi }^{2}$ statistic follows a non-central chi-square distribution with a non-centrality parameter (NCP) that scales with the effective sample size and the squared effect size. As a result, the expected value of the test statistic increases linearly with the effective sample size, making the mean ${\chi }^{2}$ statistic a useful proxy for comparing power across methods or datasets. See Supplementary Methods for further details.

### Gene-set enrichment analysis

We conducted gene-set enrichment analysis using enrichR [35] to investigate the gene ontologies enriched among genes identified by isoTWAS. The analysis queried multiple databases, including GO Biological Process 2023, GO Cellular Component 2023, GO Molecular Function 2023, KEGG 2021 Human, Reactome 2022, and ChEA 2022. ChEA is a curated database of transcription factor targets across multiple ChIP-chip, ChIP-seq, ChIP-PET and DamID assays across a host of tissues and cell-types [79]. We extracted the top 10 most significant terms from each database (FDR-adjusted *P* < 0.1) and calculated the odds ratios to identify over-represented gene functions.

### Quantitative trait locus mapping and Bayesian colocalization

For the same GTEx tissues that were used in TWAS/isoTWAS, we mapped *cis*-eQTLs for all genes and isoforms using all SNPs within a 1 Mb interval around the transcription start site. We used ordinary least squares to estimate the allelic effect of the SNP (coded as 0, 1, or 2 copies of the risk allele) on gene or isoform expression, adjusted for the same variables used in the original GTEx analyses (five principal components of the genotype matrix, 30–60 PEER factors depending on sample size for the given tissue, age at death, sex). We estimated the eQTL effect sizes, Wald-type standard errors, and *P*-values.

Next, using GWAS summary statistics for each of the 12 cancer outcomes, gene- and isoform-eQTL summary statistics, and reference LD for European ancestry individuals from the 1000 Genomes Project [76], we conducted Bayesian colocalization using eCAVIAR [36] to estimate the CoLocalization Posterior Probability (CLPP) that the same SNP is causal for both cancer risk and the gene or isoform expression. We considered a gene or isoform to colocalize with a GWAS-significant locus if three conditions were met: a SNP has a [1] strong effect on cancer risk (GWAS *P* < 5 x 10^−^^8^) [2], strong effect on gene or isoform expression (eQTL *P* < 10^−^^6^), and [3] CLPP > 0.01, as is proposed in the original eCAVIAR paper.

### Estimation of expression-mediated SNP heritability

We employed mediated expression score regression (MESC) [9] to estimate the proportion of total SNP heritability (h^2^) mediated by the *cis*-genetic component of gene or isoform expression levels (h^2^_med_). First, using the genotypes of all SNPs, expression of all genes or isoforms across all 48 tissues in GTEx, and the same covariates used in the eQTL analysis above, we estimated eQTL effect sizes in each tissue using LASSO regression (./run_mesc.py --compute-expscore-indiv…). Next, we meta-analyzed expression scores across the 49 tissues and estimated h^2^_med_ for each of the 12 cancer outcomes by using GWAS summary statistics munged to the LD score regression sumstats format. This analysis provided estimates of h^2^, h^2^_med_, and h^2^_med_/h^2^, each with standard errors and *P*-values for the hypothesis test comparing to the null value of no heritability.

## Supplementary information

Supplemental Methods, Results, Figures, Table Legends, Data Legends, and References

Supplemental Tables S1–S13.

## References

[1] Sud A, Kinnersley B, Houlston RS. Genome-wide association studies of cancer: current insights and future perspectives. Nat Rev Cancer. 2017;17:692–704. 29026206 10.1038/nrc.2017.82

[2] Zhang H, Ahearn TU, Lecarpentier J, Barnes D, Beesley J, Qi G, et al. Genome-wide association study identifies 32 novel breast cancer susceptibility loci from overall and subtype-specific analyses. Nat Genet. 2020;52:572–81. 32424353 10.1038/s41588-020-0609-2 PMC7808397

[3] Wang A, Shen J, Rodriguez AA, Saunders EJ, Chen F, Janivara R, et al. Characterizing prostate cancer risk through multi-ancestry genome-wide discovery of 187 novel risk variants. Nat Genet. 2023;55:2065–74. 37945903 10.1038/s41588-023-01534-4 PMC10841479

[4] Gamazon ER, Wheeler HE, Shah KP, Mozaffari SV, Aquino-Michaels K, Carroll RJ, et al. A gene-based association method for mapping traits using reference transcriptome data. Nat Genet. 2015;47:1091–8. 26258848 10.1038/ng.3367 PMC4552594

[5] Gusev A, Ko A, Shi H, Bhatia G, Chung W, Penninx BWJH, et al. Integrative approaches for large-scale transcriptome-wide association studies. Nat Genet. 2016;48:245–52. 26854917 10.1038/ng.3506 PMC4767558

[6] Wainberg M, Sinnott-Armstrong N, Mancuso N, Barbeira AN, Knowles DA, Golan D, et al. Opportunities and challenges for transcriptome-wide association studies. Nat Genet. 2019;51:592–9. 30926968 10.1038/s41588-019-0385-z PMC6777347

[7] Mancuso N, Gayther S, Gusev A, Zheng W, Penney KL, Kote-Jarai Z, et al. Large-scale transcriptome-wide association study identifies new prostate cancer risk regions. Nat Commun. 2018;9:4079. 30287866 10.1038/s41467-018-06302-1 PMC6172280

[8] Gao G, Fiorica PN, McClellan J, Barbeira AN, Li JL, Olopade OI, et al. A joint transcriptome-wide association study across multiple tissues identifies candidate breast cancer susceptibility genes. American J Hum Genet. 2023;110:950–62. 10.1016/j.ajhg.2023.04.005 PMC10257003 37164006

[9] Yao DW, O’Connor LJ, Price AL, Gusev A. Quantifying genetic effects on disease mediated by assayed gene expression levels. Nat Genet. 2020;52:626–33. 32424349 10.1038/s41588-020-0625-2 PMC7276299

[10] Mostafavi H, Spence JP, Naqvi S, Pritchard JK. Systematic differences in discovery of genetic effects on gene expression and complex traits. Nat Genet. 2023;55:1866–75. 37857933 10.1038/s41588-023-01529-1 PMC12270542

[11] Zhang Y, Qian J, Gu C, Yang Y. Alternative splicing and cancer: a systematic review. Sig Transduct Target Ther. 2021;6:1–14. 10.1038/s41392-021-00486-7 PMC7902610 33623018

[12] Wang E, Aifantis I. RNA Splicing and Cancer. Trends Cancer. 2020;6:631–44. 32434734 10.1016/j.trecan.2020.04.011

[13] Bhattacharya A, Vo DD, Jops C, Kim M, Wen C, Hervoso JL, et al. Isoform-level transcriptome-wide association uncovers genetic risk mechanisms for neuropsychiatric disorders in the human brain. Nat Genet. 2023;55:2117–28. 38036788 10.1038/s41588-023-01560-2 PMC10703692

[14] O’Connell KS, Koromina M, Veen T van der, Boltz T, David FS, Yang JM, et al. Genomics yields biological and phenotypic insights into bipolar disorder [Internet]. medRxiv; 2024 [cited 2024 Aug 28]. p. 2023.10.07.23296687. Available from: https://www.medrxiv.org/content/10.1101/2023.10.07.23296687v2

[15] K M, S L, J D, J B, S H, S K, et al. Association analysis identifies 65 new breast cancer risk loci. Nature [Internet]. 2017 Nov 2 [cited 2024 Oct 10];551. Available from: https://pubmed.ncbi.nlm.nih.gov/29059683/10.1038/nature24284 PMC5798588 29059683

[16] Rl M, Kb K, K M, J B, S K, S L, et al. Identification of ten variants associated with risk of estrogen-receptor-negative breast cancer. Nat. Genet. 2017 [cited 2024 Oct 10];49. Available from: https://pubmed.ncbi.nlm.nih.gov/29058716/10.1038/ng.3785 PMC5808456 29058716

[17] O’Mara TA, Glubb DM, Amant F, Annibali D, Ashton K, Attia J, et al. Identification of nine new susceptibility loci for endometrial cancer. Nat Commun. 2018;9:3166. 30093612 10.1038/s41467-018-05427-7 PMC6085317

[18] Huyghe JR, Bien SA, Harrison TA, Kang HM, Chen S, Schmit SL, et al. Discovery of common and rare genetic risk variants for colorectal cancer. Nat Genet. 2019;51:76–87. 30510241 10.1038/s41588-018-0286-6 PMC6358437

[19] McKay JD, Hung RJ, Han Y, Zong X, Carreras-Torres R, Christiani DC, et al. Large-scale association analysis identifies new lung cancer susceptibility loci and heterogeneity in genetic susceptibility across histological subtypes. Nat Genet. 2017;49:1126–32. 28604730 10.1038/ng.3892 PMC5510465

[20] Phelan CM, Kuchenbaecker KB, Tyrer JP, Kar SP, Lawrenson K, Winham SJ, et al. Identification of 12 new susceptibility loci for different histotypes of epithelial ovarian cancer. Nat Genet. 2017;49:680–91. 28346442 10.1038/ng.3826 PMC5612337

[21] Schumacher FR, Al Olama AA, Berndt SI, Benlloch S, Ahmed M, Saunders EJ, et al. Association analyses of more than 140,000 men identify 63 new prostate cancer susceptibility loci. Nat Genet. 2018;50:928–36. 29892016 10.1038/s41588-018-0142-8 PMC6568012

[22] GTEx Consortium. The GTEx Consortium atlas of genetic regulatory effects across human tissues. Science. 2020;369:1318–30. 32913098 10.1126/science.aaz1776 PMC7737656

[23] Chakravarty D, Gao J, Phillips S, Kundra R, Zhang H, Wang J, et al. OncoKB: A Precision Oncology Knowledge Base. JCO Precis Oncol 2017;1:1–16. 10.1200/PO.17.00011 PMC5586540 28890946

[24] Suehnholz SP, Nissan MH, Zhang H, Kundra R, Nandakumar S, Lu C, et al. Quantifying the Expanding Landscape of Clinical Actionability for Patients with Cancer. Cancer Discov. 2024;14:49–65. 37849038 10.1158/2159-8290.CD-23-0467 PMC10784742

[25] Carroll JS. Mechanisms of oestrogen receptor (ER) gene regulation in breast cancer. Eur J Endocrinol. 2016;175:R41–49. 26884552 10.1530/EJE-16-0124 PMC5065078

[26] Pulica R, Cohen-Solal K, Lasfar A Evaluating the Role of RUNX2 in Cancer and Its Potential as a Therapeutic Target. In: Rezaei N, editor. Handbook of Cancer and Immunology [Internet]. Cham: Springer International Publishing; 2022 [cited 2024 Aug 28]. p. 1–22. Available from: 10.1007/978-3-030-80962-1_254-1

[27] Agarwal N, Theodorescu D. The Role of Transcription Factor YY1 in the Biology of Cancer. Crit Rev Oncog. 2017;22:13–21. 29604933 10.1615/CritRevOncog.2017021071 PMC8788901

[28] Zeng T, Spence JP, Mostafavi H, Pritchard JK. Bayesian estimation of gene constraint from an evolutionary model with gene features. Nat Genet. 2024;56:1632–43. 38977852 10.1038/s41588-024-01820-9

[29] Chen H, Liu H, Qing G. Targeting oncogenic Myc as a strategy for cancer treatment. Sig Transduct Target Ther. 2018;3:1–7. 10.1038/s41392-018-0008-7 PMC5837124 29527331

[30] Paller CJ, Tukachinsky H, Maertens A, Decker B, Sampson JR, Cheadle JP, et al. Pan-Cancer Interrogation of MUTYH Variants Reveals Biallelic Inactivation and Defective Base Excision Repair Across a Spectrum of Solid Tumors. JCO Precis Oncol. 2024;8:e2300251. 10.1200/PO.23.00251 PMC10901435 38394468

[31] Gupta SK, Gallego C, Johnson GL, Heasley LE. MAP kinase is constitutively activated in gip2 and src transformed rat 1a fibroblasts. Journal Biol Chem. 1992;267:7987–90. 1314814

[32] Ikezu T, Okamoto T, Murayama Y, Okamoto T, Homma Y, Ogata E, et al. Bidirectional regulation of c-fos promoter by an oncogenic gip2 mutant of G alpha i2. A novel implication of retinoblastoma gene product. Journal Biol Chem. 1994;269:31955–61. 7989371

[33] Wang Z, Zheng W, Chen Z, Wu S, Chang H, Cai M, et al. Pan-Cancer analysis shows that ACO2 is a potential prognostic and immunotherapeutic biomarker for multiple cancer types including hepatocellular carcinoma. Front Oncol. 2022;12:1055376. 36531056 10.3389/fonc.2022.1055376 PMC9748622

[34] Xu J, Li L, Shi P, Cui H, Yang L. The Crucial Roles of Bmi-1 in Cancer: Implications in Pathogenesis, Metastasis, Drug Resistance, and Targeted Therapies. Int J Mol Sci. 2022;23:8231. 35897796 10.3390/ijms23158231 PMC9367737

[35] Chen EY, Tan CM, Kou Y, Duan Q, Wang Z, Meirelles GV, et al. Enrichr: interactive and collaborative HTML5 gene list enrichment analysis tool. BMC Bioinforma. 2013;14:128. 10.1186/1471-2105-14-128 PMC3637064 23586463

[36] Hormozdiari F, van de Bunt M, Segrè AV, Li X, Joo JWJ, Bilow M, et al. Colocalization of GWAS and eQTL Signals Detects Target Genes. Am J Hum Genet. 2016;99:1245–60. 27866706 10.1016/j.ajhg.2016.10.003 PMC5142122

[37] Chen H, Majumdar A, Wang L, Kar S, Brown KM, Feng H, et al. Large-scale cross-cancer fine-mapping of the 5p15.33 region reveals multiple independent signals. HGG Adv. 2021;2:100041. 34355204 10.1016/j.xhgg.2021.100041 PMC8336922

[38] Bau DT, Tsai CW, Chang WS, Yang JS, Liu TY, Lu HF, et al. Genetic susceptibility to prostate cancer in Taiwan: A genome-wide association study. Mol Carcinog. 2024;63:617–28. 38390760 10.1002/mc.23676

[39] Bai J, Zhao Y, Shi K, Fan Y, Ha Y, Chen Y, et al. HIF-1α-mediated LAMC1 overexpression is an unfavorable predictor of prognosis for glioma patients: evidence from pan-cancer analysis and validation experiments. Journal Transl Med. 2024;22:391. 38678297 10.1186/s12967-024-05218-3 PMC11056071

[40] Kunitomi H, Kobayashi Y, Wu RC, Takeda T, Tominaga E, Banno K, et al. LAMC1 is a prognostic factor and a potential therapeutic target in endometrial cancer. J Gynecol Oncol. 2019;31:e11. 31912669 10.3802/jgo.2020.31.e11 PMC7044014

[41] Bogdanova N, Helbig S, Dörk T. Hereditary breast cancer: ever more pieces to the polygenic puzzle. Hereditary Cancer Clin Pract. 2013;11:12. 10.1186/1897-4287-11-12 PMC3851033 24025454

[42] Antoniou AC, Wang X, Fredericksen ZS, McGuffog L, Tarrell R, Sinilnikova OM, et al. A locus on 19p13 modifies risk of breast cancer in BRCA1 mutation carriers and is associated with hormone receptor-negative breast cancer in the general population. Nat Genet. 2010;42:885–92. 20852631 10.1038/ng.669 PMC3130795

[43] Bolton KL, Tyrer J, Song H, Ramus SJ, Notaridou M, Jones C, et al. Common variants at 19p13 are associated with susceptibility to ovarian cancer. Nat Genet. 2010;42:880–4. 20852633 10.1038/ng.666 PMC3125495

[44] Jaganathan K, Kyriazopoulou Panagiotopoulou S, McRae JF, Darbandi SF, Knowles D, Li YI, et al. Predicting Splicing from Primary Sequence with Deep Learning. Cell. 2019;176:535–48.e24. 30661751 10.1016/j.cell.2018.12.015

[45] Frankish A, Diekhans M, Ferreira AM, Johnson R, Jungreis I, Loveland J, et al. GENCODE reference annotation for the human and mouse genomes. Nucleic Acids Res. 2019;47:D766–73. 30357393 10.1093/nar/gky955 PMC6323946

[46] ENCODE Project Consortium. An integrated encyclopedia of DNA elements in the human genome. Nature. 2012;489:57–74. 22955616 10.1038/nature11247 PMC3439153

[47] Li YI, Knowles DA, Humphrey J, Barbeira AN, Dickinson SP, Im HK, et al. Annotation-free quantification of RNA splicing using LeafCutter. Nat Genet. 2018;50:151–8. 29229983 10.1038/s41588-017-0004-9 PMC5742080

[48] Kundaje A, Meuleman W, Ernst J, Bilenky M, Yen A, Heravi-Moussavi A, et al. Integrative analysis of 111 reference human epigenomes. Nature. 2015;518:317–30. 25693563 10.1038/nature14248 PMC4530010

[49] Zeng T, Li YI. Predicting RNA splicing from DNA sequence using Pangolin. Genome Biol. 2022;23:103. 35449021 10.1186/s13059-022-02664-4 PMC9022248

[50] Backman JD, Li AH, Marcketta A, Sun D, Mbatchou J, Kessler MD, et al. Exome sequencing and analysis of 454,787 UK Biobank participants. Nature. 2021;599:628–34. 34662886 10.1038/s41586-021-04103-z PMC8596853

[51] Yandell M, Huff C, Hu H, Singleton M, Moore B, Xing J, et al. A probabilistic disease-gene finder for personal genomes. Genome Res. 2011;21:1529–42. 21700766 10.1101/gr.123158.111 PMC3166837

[52] Hu H, Huff CD, Moore B, Flygare S, Reese MG, Yandell M. VAAST 2.0: Improved Variant Classification and Disease-Gene Identification Using a Conservation-Controlled Amino Acid Substitution Matrix. Genet Epidemiol. 2013;37:622–34. 23836555 10.1002/gepi.21743 PMC3791556

[53] Wang X, Goldstein DB. Enhancer Domains Predict Gene Pathogenicity and Inform Gene Discovery in Complex Disease. Am J Hum Genet. 2020;106:215–33. 32032514 10.1016/j.ajhg.2020.01.012 PMC7010980

[54] McKay JD, Hung RJ, Gaborieau V, Boffetta P, Chabrier A, Byrnes G, et al. Lung cancer susceptibility locus at 5p15.33. Nat Genet. 2008;40:1404–6. 18978790 10.1038/ng.254 PMC2748187

[55] Rafnar T, Sulem P, Stacey SN, Geller F, Gudmundsson J, Sigurdsson A, et al. Sequence variants at the TERT-CLPTM1L locus associate with many cancer types. Nat Genet. 2009;41:221–7. 19151717 10.1038/ng.296 PMC4525478

[56] Shete S, Liu H, Wang J, Yu R, Sturgis EM, Li G, et al. A Genome-Wide Association Study Identifies Two Novel Susceptible Regions for Squamous Cell Carcinoma of the Head and Neck. Cancer Res. 2020;80:2451–60. 32276964 10.1158/0008-5472.CAN-19-2360 PMC7299763

[57] Lesseur C, Diergaarde B, Olshan AF, Wünsch-Filho V, Ness AR, Liu G, et al. Genome-wide association analyses identify new susceptibility loci for oral cavity and pharyngeal cancer. Nat Genet. 2016;48:1544–50. 27749845 10.1038/ng.3685 PMC5131845

[58] Petersen GM, Amundadottir L, Fuchs CS, Kraft P, Stolzenberg-Solomon RZ, Jacobs KB, et al. A genome-wide association study identifies pancreatic cancer susceptibility loci on chromosomes 13q22.1, 1q32.1 and 5p15.33. Nat Genet. 2010;42:224–8. 20101243 10.1038/ng.522 PMC2853179

[59] James MA, Vikis HG, Tate E, Rymaszewski AL, You M. CRR9/CLPTM1L regulates cell survival signaling and is required for Ras transformation and lung tumorigenesis. Cancer Res. 2014;74:1116–27. 24366883 10.1158/0008-5472.CAN-13-1617 PMC6005686

[60] Parashar D, Geethadevi A, McAllister D, Ebben J, Peterson FC, Jensen DR, et al. Targeted biologic inhibition of both tumor cell-intrinsic and intercellular CLPTM1L/CRR9-mediated chemotherapeutic drug resistance. npj Precis Onc. 2021;5:1–13. 10.1038/s41698-021-00152-9 PMC7925570 33654182

[61] Kim NW, Piatyszek MA, Prowse KR, Harley CB, West MD, Ho PL, et al. Specific association of human telomerase activity with immortal cells and cancer. Science. 1994;266:2011–5. 7605428 10.1126/science.7605428

[62] Ge M, Shi M, An C, Yang W, Nie X, Zhang J, et al. Functional evaluation of TERT-CLPTM1L genetic variants associated with susceptibility of papillary thyroid carcinoma. Sci Rep. 2016;6:26037. 27185198 10.1038/srep26037 PMC4869017

[63] Gholami M, Zoughi M, Behboo R, Taslimi R, Kazemeini A, Bastami M, et al. Association of miRNA targetome variants in LAMC1 and GNB3 genes with colorectal cancer and obesity. Cancer Med. 2022;11:3923–38. 35373932 10.1002/cam4.4713 PMC9636511

[64] Hazelwood E, Canson DM, Wang X, Kho PF, Legge D, Constantinescu AE, et al. Integrating multi-tissue expression and splicing data to prioritise anatomical subsite- and sex-specific colorectal cancer susceptibility genes with therapeutic potential [Internet]. medRxiv; 2024 [cited 2024 Oct 14]. p. 2024.09.10.24313450. Available from: https://www.medrxiv.org/content/10.1101/2024.09.10.24313450v1

[65] Qin Y, Rodin S, Simonson OE, Hollande F. Laminins and cancer stem cells: Partners in crime?. Semin Cancer Biol. 2017;45:3–12. 27491691 10.1016/j.semcancer.2016.07.004

[66] Galatenko VV, Maltseva DV, Galatenko AV, Rodin S, Tonevitsky AG. Cumulative prognostic power of laminin genes in colorectal cancer. BMC Med Genomics. 2018;11:9. 29504916 10.1186/s12920-018-0332-3 PMC5836818

[67] Mancuso N, Freund MK, Johnson R, Shi H, Kichaev G, Gusev A, et al. Probabilistic fine-mapping of transcriptome-wide association studies. Nat Genet. 2019;51:675–82. 30926970 10.1038/s41588-019-0367-1 PMC6619422

[68] Bhattacharya A, Hirbo JB, Zhou D, Zhou W, Zheng J, Kanai M, et al. Best practices for multi-ancestry, meta-analytic transcriptome-wide association studies: Lessons from the Global Biobank Meta-analysis Initiative. Cell Genomics. 2022;2:100180. 36341024 10.1016/j.xgen.2022.100180 PMC9631681

[69] Kachuri L, Mak ACY, Hu D, Eng C, Huntsman S, Elhawary JR, et al. Gene expression in African Americans, Puerto Ricans and Mexican Americans reveals ancestry-specific patterns of genetic architecture. Nat Genet. 2023;55:952–63. 37231098 10.1038/s41588-023-01377-z PMC10260401

[70] Bhattacharya A, García-Closas M, Olshan AF, Perou CM, Troester MA, Love MI. A framework for transcriptome-wide association studies in breast cancer in diverse study populations. Genome Biol. 2020;21:42. 32079541 10.1186/s13059-020-1942-6 PMC7033948

[71] Keys KL, Mak ACY, White MJ, Eckalbar WL, Dahl AW, Mefford J, et al. On the cross-population generalizability of gene expression prediction models. PLoS Genet. 2020;16:e1008927. 32797036 10.1371/journal.pgen.1008927 PMC7449671

[72] Patro R, Duggal G, Love MI, Irizarry RA, Kingsford C. Salmon provides fast and bias-aware quantification of transcript expression. Nat Methods. 2017;14:417–9. 28263959 10.1038/nmeth.4197 PMC5600148

[73] Love MI, Soneson C, Hickey PF, Johnson LK, Pierce NT, Shepherd L, et al. Tximeta: Reference sequence checksums for provenance identification in RNA-seq. PLOS Computational Biol. 2020;16:e1007664. 10.1371/journal.pcbi.1007664 PMC7059966 32097405

[74] Ritchie ME, Phipson B, Wu D, Hu Y, Law CW, Shi W, et al. limma powers differential expression analyses for RNA-sequencing and microarray studies. Nucleic Acids Res. 2015;43:e47. 25605792 10.1093/nar/gkv007 PMC4402510

[75] Love MI, Huber W, Anders S. Moderated estimation of fold change and dispersion for RNA-seq data with DESeq2. Genome Biol. 2014;15:550. 25516281 10.1186/s13059-014-0550-8 PMC4302049

[76] Auton A, Abecasis GR, Altshuler DM, Durbin RM, Abecasis GR, Bentley DR, et al. A global reference for human genetic variation. Nature. 2015;526:68–74. 26432245 10.1038/nature15393 PMC4750478

[77] Lindström S, Wang L, Feng H, Majumdar A, Huo S, Macdonald J, et al. Genome-wide analyses characterize shared heritability among cancers and identify novel cancer susceptibility regions. JNCI: J Natl Cancer Inst. 2023;115:712–32. 36929942 10.1093/jnci/djad043 PMC10248849

[78] Bhattacharya A isoTWAS models for 48 GTEx models (04/2024) [Internet]. Zenodo; 2024 [cited 2024 Sep 10]. Available from: https://zenodo.org/records/11048201

[79] Lachmann A, Xu H, Krishnan J, Berger SI, Mazloom AR, Ma’ayan A. ChEA: transcription factor regulation inferred from integrating genome-wide ChIP-X experiments. Bioinformatics. 2010;26:2438. 20709693 10.1093/bioinformatics/btq466 PMC2944209

